# Epigenetic Modifications of Major Depressive Disorder

**DOI:** 10.3390/ijms17081279

**Published:** 2016-08-05

**Authors:** Kathleen Saavedra, Ana María Molina-Márquez, Nicolás Saavedra, Tomás Zambrano, Luis A. Salazar

**Affiliations:** 1 Center of Molecular Biology and Pharmacogenetics, Scientific and Technological Bioresource Nucleus, Universidad de La Frontera, Temuco 4811230, Chile; kathleen.saavedra@ufrontera.cl (K.S.); anamariamolinamarquez@gmail.com (A.M.M.-M.); nicolas.saavedra@ufrontera.cl (N.S.); tomas.zambrano@ufrontera.cl (T.Z.); 2 Millennium Institute for Research in Depression and Personality (MIDAP), Universidad de La Frontera, Temuco 4811230, Chile

**Keywords:** major depressive disorder, depression, epigenetic modifications, methylation, histone modification, miRNAs, biomarkers

## Abstract

Major depressive disorder (MDD) is a chronic disease whose neurological basis and pathophysiology remain poorly understood. Initially, it was proposed that genetic variations were responsible for the development of this disease. Nevertheless, several studies within the last decade have provided evidence suggesting that environmental factors play an important role in MDD pathophysiology. Alterations in epigenetics mechanism, such as DNA methylation, histone modification and microRNA expression could favor MDD advance in response to stressful experiences and environmental factors. The aim of this review is to describe genetic alterations, and particularly altered epigenetic mechanisms, that could be determinants for MDD progress, and how these alterations may arise as useful screening, diagnosis and treatment monitoring biomarkers of depressive disorders.

## 1. Introduction

Major depressive disorder (MDD) is a chronic and debilitating disease that affects more than 350 million people worldwide, making it one of the most common mental illnesses [1]. MDD ranks second in terms of disease burden, accounting for 40.5% of disability-adjusted life years [2]. The World Health Organization (WHO) has estimated that MDD will be the second leading cause of disability throughout the world, preceded only by ischemic heart disease [3]. These data include MDD among major public health problems.

MDD is defined by the fifth edition of the Diagnostic and Statistical Manual of Mental Disorders (DSM-V) as a complex and heterogeneous syndrome that covers a wide spectrum of symptoms, including anhedonia, disturbed sleep, reduced appetite and energy, depressed mood, reduced concentration and suicidal thoughts, among others [4,5,6]. Since MDD diagnosis continues to be based in detecting such clinical symptoms, current research efforts aim to identify specifics biomarkers that might facilitate depressive disorders diagnosis. However, such efforts have been unsuccessful, partly because depressive disorders neurobiological basis and pathophysiology remain poorly understood. Genetic factors, and their association with the environment, play important roles in MDD; therefore, exploring the genetic background might reveal important information about the mechanisms underlying MDD development.

The present review describes how genetics and epigenetics alterations can be important determinants for MDD progress, and how these alterations may arise as interesting biomarkers for screening, diagnosis and treatment monitoring of depressive disorders.

## 2. Genetics of Major Depression

Epidemiological studies have shown that major depression is a familial disorder. A meta-analysis derived from five twin studies including more than 21,000 subjects revealed a genetic contribution for MDD development of 37% (95% CI: 31%–42%), added to evidence obtained from family studies showing two- to three-fold increased MDD risk (Mantel–Haenszel odds ratio = 2.84, 95% CI = 2.31–3.49) during the lifetime among first-degree relatives [7]. Moreover, when considering disease severity, defined by relapse rate and early disease onset, depression heritability may increase by up to 70% [8,9]. The important heritability observed in this disorder has elevated the expectations of identifying key genes involved in MDD progress that might be considered potentials risk indicators. However, no specific genetic variants have been identified as robust contributors to major depression. Several linkage and association studies have been conducted with the hope to identify risk-associated genes. Both are complementary methods to locate susceptibility genes, but they have failed to identify universal genetic risk or causal factors for depression disorders. At present, and due to the rapid development of technological advances in the field of genomics, it is possible to perform large genome-wide association studies (GWAS). This method, although combining the advantage of the breadth of linkage with the power of association, has also failed, as few genetic variants have been strongly implicated in depressive disorders. Lee et al. [10] performed a meta-analysis including 4346 cases and 4430 controls, and found that genes involved in glutamatergic synaptic neurotransmission were significantly associated with MDD. However, they were not able to associate a genetic variant with MDD risk. Wray et al. [11] studied 5763 cases and 6901 controls, showing evidence of an association between MDD and polymorphisms at or near genes with plausible biological relevance, such as galanin (*GAL*) and adenylate cyclase 3 (*ADCY3*). Ripke et al. performed a meta-analysis from 9240 cases and 9519 controls, with replication in 6783 cases and 50,695 controls, but no genetics variants achieved genome-wide significance, neither in the MDD discovery phase nor in the MDD replication phase [11,12]. Despite these negative results, alterations in numerous genes have been linked to depression pathophysiology in different studies. Some of these genes include regulators of neurotransmitter signaling as serotonin transporter (*SLC6A4*; particularly the *5-HTTLPR* polymorphism), monoamine oxidase A (*MAOA*), catechol-o-methyltransferase (*COMT*), regulators of neural plasticity and connectivity as the brain-derived neurotrophic factor (*BDNF*) and the enzyme tryptophan hydroxylase (*TPH*) that mediates serotonin synthesis on peripheral (*TPH1*) and cerebral (*TPH2*) level, among others. Studies of polymorphisms associated with these genes have showed that genetic variants might increase genetic susceptibility to develop depression, anxiety, stress or cognitive functions alterations [13,14,15,16,17,18,19,20,21]. However, again, these results lack consistency and have not shown the desired reproducibility. 

The discrepancy observed between evidence showing MDD as a family disease together with the impossibility to identify genetic alterations associated with MDD suggest that additional factors are involved in MDD development. In the report of Sullivan et al. [7], they demonstrated that common environmental influences had a minimal contribution of 0% (95% CI: 0%–5%), while individual-specific environmental factors showed a significant contribution of 63% (95% CI: 58%–67%). Since MDD cannot be attributed to a single genetic mutation or exposure to one specific environmental stimulus, MDD is proposed to arise from an interaction between genetic variations and environmental factors [22,23]. Regarding environmental factors influencing MDD development, exposure to environmental stressors, especially as traumatic events in early life, is one of the strongest risk factors described to date. Recently, it has been suggested that adverse environmental stimulus can stably alter gene expression in healthy subjects and encourage depression development through epigenetic mechanisms [24,25]. Moreover, reports show that epigenetic processes would be involved in the development of several human diseases, including psychiatric disorders as MDD [26].

## 3. Epigenetic Modifications and Depressive Disorders

Epigenetics refers to changes in gene expression that are not due to alterations in DNA sequence; these changes can be potentially heritable, but environmentally modifiable, and could explain different scenarios in which medical observations confront traditional genetics [27,28]. Epigenetic regulation is fundamental for many cellular processes including gene (mRNA) and microRNA (miRNA) expression, DNA–protein interactions, suppression of transposable elements, cellular differentiation, embryogenesis, X-chromosome inactivation and genomic imprinting. In the same way, epigenetic regulation not only regulates physiological but also pathological processes [29]. In fact, has been described that the epigenetics has an important role in the development of many mental illness, such as MDD [30,31].

Overall, epigenetic modifications can be grouped into three general categories: DNA methylation, histone modification and nucleosome positioning. Non-coding RNA (ncRNA)-mediated regulation is also considered an important epigenetic regulation in the pathophysiologic process of depression [32,33].

### 3.1. DNA Methylation

The most widely studied epigenetic modification in humans is DNA methylation. This mechanism consist in the addition of a methyl group at the 5′ position of cytosines in cytosine-phosphate-guanine dinucleotides (CpG), a process catalyzed by DNA methyltransferases (DNMTs) occurring almost exclusively in CpG dinucleotides usually clustered within the promoter region of genes, termed CpG islands [29]. Cytosine methylation reduces the access of transcription factors into regulatory elements; therefore, DNA methylation is associated with transcriptional repression. Evidence suggests that DNA methylation is responsive to environmental signals [34], and recently, a large number of studies conducted in animal models and humans support the idea that DNA methylation plays an important role in mediating stress effects.

**Figure 1:**
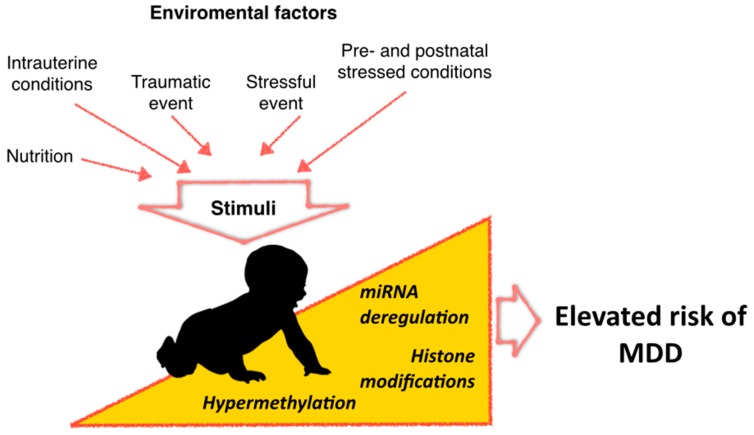
Demanding conditions in utero or during the first years can elevate the risk of both neurological and psychiatric disorders, possibly by mechanisms involving epigenetic dysregulation.

Important environmental factors to consider in the risk for developing MDD occur early during the gestational stage of an individual [35]. Accordingly, gestational stimuli play important roles in the development of various neuropsychiatric disorders, including MDD [36,37]. Intrauterine conditions can have long-term effects in terms of risk of neurological or psychiatric disorders, which would be mediated through epigenetic modifications such as DNA methylation (Figure 1).

Nieratschker et al. [38] reported that *MORC1* methylation, a gene that evokes a depression-like phenotype in mice, is a candidate marker for MDD development associated with early life stress in rodents, primates and humans undergoing prenatal stressed conditions [39,40]. Interesting models for the study of variations in DNA methylation profiles are the monozygotic twins, as they have almost the same DNA sequence, but frequently show phenotypic discordance [41,42,43,44,45]. Monozygotic twins methylation profile can be very similar, not only by the nearly identical DNA sequence that they possess, but also because both individuals are subjected to one common pre- and post-natal environment [46]. However, there are still differences in methylation profiles, which can be produced by exposure to environmental causes influencing one of the twins, or by stochastic factors [47,48]. Considering the aforementioned background, Córdova-Palomera et al. [49] evaluated differences in DNA methylation of monozygotic twins using two analytical strategies to identify differentially methylated probes (DMPs) and variably methylated probes (VMPs), showing associations with differences in the psychopathological status of twins. Most DMPs were located in genes previously related to neuropsychiatric disorders; one of these was the WD Repeat Domain 26 (*WDR26*) gene, implicated in MDD from GWAS data [11,50]. VMPs were also located in genes such as Calcium Channel, Voltage-Dependent, L Type, Alpha 1C (*CACNA1C*), Insulin-Like Growth Factor *2* (*IGF2*) and the p38 MAP kinase (*MAPK11*), showing enrichment for biological processes such as glucocorticoid signaling. DNA sequence variation of *CACNA1C* has been recognized as a susceptibility factor for depressive psychopathology development, and its methylation changes have been associated with risk factors for depressive disorders as early-life stress [11,51,52]. Additionally, the activity of MAPK11 has been associated with depression phenotypes [53].

While GWAS have failed in identifying sequence variations influencing MDD susceptibility, epigenetic marks such as DNA methylation have emerged as better candidates to be employed as depression biomarkers. Sabunciyan et al. [54] performed the first genome-wide DNA methylation (NMD) scan in MDD. In that study, 39 post-mortem frontal cortex MDD samples were compared to 26 controls using the Comprehensive High-throughput Arrays for Relative Methylation (CHARM) platform, covering 3.5 million CpGs. They identified 224 candidate regions having DNA methylation differences >10% in highly enriched regions for neuronal growth and development genes. Further experimental validation showed the greatest differences in Proline Rich Membrane Anchor 1 (*PRIMA1*), with 12%–15% increased DNA methylation in MDD individuals than controls [54]. *PRIMA1* is important within MDD biology as it encodes a protein that organizes acetylcholinesterase (AChE) into tetramers, anchoring AChE to neural cell membranes [55,56].

Postmortem analysis from brain tissue of individuals with neuropsychiatric disorders have shown that DNA methylation is compromised in comparison to control individuals [27]. Brain tissue is considered an ideal sample for DNA methylation analyses in neuropsychiatric disorders. However, its accessibility is highly difficult; therefore, tissue sampling is restricted to postmortem collection. This critical issue drives the need to search for additional non-invasive samples with better accessibility that also reflects the biochemical and molecular changes occurring in the brain. Thus, numerous studies using peripheral blood as a non-invasive model for DNA methylation analyses in neuropsychiatric diseases have been performed, allowing the identification of potential circulating biomarkers for MDD diagnosis. An example is the study performed by Numata et al. [57], identifying DNA methylation markers able to distinguish between medication-free patients with MDD and non-psychiatric controls. In this study, significant diagnostic differences in DNA methylation were observed at 363 CpG sites, all of them demonstrating lower DNA methylation in patients with MDD than controls [57]. Some of these markers were Cell Cycle Associated Protein 1 (*CAPRIN1*), cAMP-response element binding protein/p300-interacting transactivator with Glu/Asp-rich carboxy-terminal domain-2 (*CITED2*), Diacylglycerol Kinase (*DGKH*), Glycogen Synthase Kinase 3 Beta (*GSK3B*) and Serum/Glucocorticoid Regulated Kinase 1 (*SGK1*), genes previously associated with MDD [40,58,59,60,61,62]. Moreover, it has been reported that DNA methylation status obtained from blood samples shows a correlation with the methylation status observed in post-mortem brain tissue. Stenz et al. [63] described a correlation between promoter methylation of Brain-Derived Neurotrophic Factor (*BDNF*) gene in blood and post-mortem brain tissue from depressed patients (Pearson, *n* = 98, 263 *r* = 0.48, *p* = 4.5 × 10^−7^). *BDNF* promotes proliferation, differentiation and survival of neurons and is crucial for neural plasticity and cognitive function [64]. Meanwhile, Januar et al. [65] proposed the detection of *BDNF* methylation in oral tissue as a potential depression biomarker (promoter I, Δmean = 0.4%, *p* = 0.0002).

Taken together, DNA methylation can be one of the several epigenetic mechanisms by which stressors can have long-term effects through gene expression alteration of exposed individuals, influencing or determining the course of depressive disorders. Numerous studies have reported the identification of genes frequently methylated and related to depressive disorder pathophysiology in peripheral blood samples, allowing potential identification of biomarkers for MDD early detection and diagnosis. A list of these biomarkers is displayed in Table 1.

### 3.2. Histone Modification

All histones undergo post-transcriptional modifications affecting the histone tail. Modifications include acetylation, methylation, phosphorylation, ubiquitination, SUMOylation and ADP-ribosylation, among others [29], and can change the DNA–histone core interaction, which is involved in gene expression regulation by chromatin remodeling. Histone acetylation involves the transferring of an acetyl group to histone tails by Histone acetyltransferases (HATS) enzymes. This process promotes histone units unfolding and chromatin decondensation, allowing transcription factors binding to genomic DNA and therefore, promoting gene expression. Conversely, enzymes known as histone deacetylases (HDACs) remove the acetyl group from the histone tail, causing chromatin condensation and preventing transcription factors access to genomic DNA, thus, decreasing gene expression [70].

Few studies investigating the effect of histone modification on depressive disorders development have been conducted (Table 2). Interestingly, the initial findings were achieved by using histone deacetylase inhibitors (HDACi), alone or in combination with antidepressants, in a variety of animal models [71,72,73,74]. An example is valproate, an HDACi commonly used as a mood stabilizer in bipolar disorder which function may be mediated through HDACs inhibition [75,76]. Thus, HDACs dysfunction can be involved in the pathophysiology of mood disorders. Covington et al. [71] explored the impact of chronic stress on histone acetylation in the nucleus accumbens (NAc), an important limbic brain region, in a chronic stress defeat model and postmortem tissue of depressed individuals. In this study, histone acetylation (H3K14ac) was transiently decreased and then stably increased in the NAc of mice after chronic social defeat stress, which was correlated with a reduction in HDAC2 levels and reproduced in postmortem tissue of depressive patients. Later, the effect of direct MS-275 infuse (a selective inhibitor of class I HDACs) in the NAc was also evaluated, resulting in a robustly antidepressant effect of chronic defeat stress on global patterns of gene expression, and suggesting that histone acetylation has an adaptive role in stress and depression [25]. HDAC5 expression studies in the NAc of mice susceptible to chronic social defeat stress have also been performed, finding that HDAC5 was repressed, whereas imipramine treatment (a chronic antidepressant) increased HDAC5 expression [77]. In addition, mice lacking HDAC5 exhibited increased depressive-like behaviors after chronic social defeat stress compared to control animals [77]. The results reported by Convington et al. [71] and Renthal et al. [77] suggest that histone modifications by HDACs play an adaptive role in chronic psychiatric illnesses response, and that HDAC5 targets may have a depressive role, while targets of HDAC2 may have an antidepressant role.

**Figure 2:**
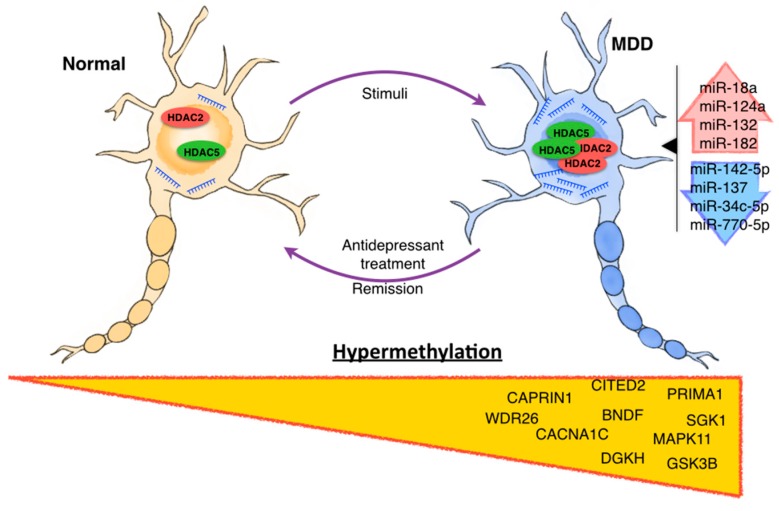
Epigenetic alterations in major depressive disorder. Evidence from human and animal observations indicates that environmental stimuli, such as stressful experiences, are associated with a deregulation of epigenetic modifications. These modifications include deregulation in profiles of hypermethylated genes, miRNA profiles and histone modifications. In addition, it has been reported that antidepressant treatment may act via modulation of these epigenetic modifications, particularly histone modification. Likewise, during recessive episodes, epigenetic modification described in MDD patients can return to their baseline state (control patients without MDD).

Other studies evaluated gene expression of the histone acetylation machinery as potential biomarkers in peripheral blood cells of depressed patients. Hobara et al. [78] assessed gene expression of 11 HDACs (including HDAC2 and -5) in peripheral white blood cells of MDD and bipolar disorder (BPD) patients during depressive and remissive episodes. Experiments revealed that HDAC2 and HDAC5 expression was significantly increased in MDD patients in the depressive state compared to controls subjects (HDAC2 *p* < 0.001; HDAC5 *p* = 0.001), while during remissive state, expression of the same HDACs was comparable to controls subjects (HDAC2 *p* = 0.975; HDAC5 *p* = 0.506), suggesting a state-dependent alteration (Figure 2) [78]. These results are consistent with those previously reported by Iga et al. [79] in peripheral leucocytes of drug-free depressive patients, in which the expression of HDAC5 was higher compared to controls. 

On the other hand, due to the trimethylated forms of histone H3, lysines (K4, K9, and K2) can serve to distinguish between active/inactive chromatin, and remain stable during tissue autolysis, investigations performed on these epigenetic marks have raised special interest within postmortem research [81]. Consequently, one of the best histone modifications studied so far corresponds to the tri-methylation of the fourth lysine tail on histone 3 (H3K4me3) [82]. This modification opens chromatin, allowing the transcriptional machinery binding to the promoter region of genes, inducing transcription initiation [65]. Enrichments of this marker are highly associated with increased gene expression levels [83,84]. Cruceanu et al. [80] analyzed the expression of transcript variants for the three synapsin genes and investigated their relationship with H3K4me3 promoter enrichment in post-mortem brain samples from BPD (*n* = 13), MDD (*n* = 18) and controls (*n* = 14) patients with no psychiatric history. The *SYN1*, *SYN2* and *SYN3* genes encode neuronal phosphoproteins belonging to the synapsin family, which are reported to play crucial activities in different neuropsychiatric disorders [85,86,87]. Cruceanu and colleagues found that histone modification markers were significantly increased in MDD, and this effect was correlated with a significant increase in *SYN2* gene expression [80]. 

### 3.3. Non-Coding RNAs

The term ncRNAs indicates different classes of RNAs that are not translated into a protein, but exert a functional role as well. There are five classes of ncRNAs distinguished so far: microRNAs (miRs), small nucleolar RNAs (snoRNAs), large intergenic non-coding RNAs (lincRNAS), PIWI-interacting RNAs (piRNAs) and transcribed ultraconserved regions (T-UCRs). However, the most widely studied class of ncRNAs corresponds to microRNAs (miRs), small ncRNAs of 22 nucleotides that mediates post-transcriptional gene silencing by controlling the translation of mRNA [88,89]. These ncRNAs are involved in many different regulatory processes, including proliferation, differentiation, apoptosis and development. They can regulate one particular target or may regulate the expression of hundreds of genes simultaneously [90]. miRs modulate mRNA expression depending on the number of mismatches between its own sequence and the sequence of the target mRNA, regulating expression by enzymatic target degradation or by preventing mRNA translation into protein due to steric hindrance of the protein synthesis machinery [91,92,93].

In recent years, the role of miRs in neuropsychiatric and neurodegenerative diseases development has gained significant attention, however, the implication of this kind of ncRNA in affective diseases, particularly MDD, is less clear. Nevertheless, recent studies have suggested that miRs play a key role in MDD pathophysiology, particularly at neurogenesis, synaptic plasticity and regulation of key genes that are critical components of signaling pathways involved in MDD. Similarly, various researches have shown miR profiles dysregulation when comparing depressed patients with normal controls (Figure 2). Table 3 shows a summary of miRs studies in depressive disorders patients.

Uchida et al. [94] reported that miR-18a inhibits translation of the glucocorticoid receptor in neuron cell culture, and that its expression in the hypothalamic paraventricular nucleus is increased in F344 rats compared to Sprague-Dawley control rats, both with repeated restraint stress. This finding could explain, at least in part, the decreased expression of glucocorticoid receptors in depressed individuals, and why the majority of depressed patients would have elevated cortisol levels in plasma and cerebrospinal fluid (CSF) [95]. Vreugdenhil et al. [96] reported that miR-18a and miR-124a decreased glucocorticoid receptor protein expression by luciferase reporter assays in NS1 cells, confirming the results obtained by Uchida et al. [94].

Dwivedi et al. [97] were the first to examine global expression patterns of miRs in the dorsolateral prefrontal cortex (dlPFC) of depressed subjects. They found 21 miRs significantly downregulated in the prefrontal cortex of depressed patients compared to normal controls, many of them implicated in cellular growth and differentiation and showing high synaptic enrichment [97,98,99].

miRs regulation of *BDNF*, a critical gene for MDD physiopathology, has also been evaluated in serum of MDD patients. Li et al. [33] found two miRs (miR-182 and miR-132) as putative regulators of *BDNF* expression in MDD patients. In that study, miR-182 and miR-132 levels were upregulated, while the expression of *BDNF* was repressed. The Self-Rating Depression Scale score showed an inverse correlation with serum BDNF levels, while demonstrating a direct correlation with miR-132 levels [33]. In addition, Smalheiser et al. [98] demonstrated that miR-494 and miR-335 are downregulated in the prefrontal cortex of depressed suicide patients [98]. 

Antidepressant (AD) treatment affects miRs expression, allowing the identification of new miRs involved in MDD physiopathology. Bocchio-Chiavetto et al. [100] conducted a whole-miRNome quantitative analysis using qRT-PCR and evaluating miRs expression changes in the blood of 10 depressed subjects following 12 weeks of escitalopram treatment. Thirty miRs were differentially expressed after escitalopram treatment: 28 miRs were upregulated and two miRs were strongly downregulated. Among these miRs differentially regulated, miR-132 has been implicated in both neurogenesis and synaptic plasticity, whereas miR-26a, miR26b and miR-183 contribute to BDNF function in the brain [100,101].

The use of circulating miRs as potential clinical biomarkers has gained substantial interest in recent years. Numerous studies indicate that miRs can be detected in several body fluids, such as blood and cerebrospinal fluid, in addition to highlighting its great stability [32,102,103,104,105,106]. Other studies show that miRs, under health conditions, have a stable expression; while pathological conditions within the central nervous system can alter their expression greatly [107]. Changes in circulating miRs correlate with expression changes of miRs evaluated in neuronal tissues [108,109,110]. In this context, Belzeaux et al. [96] evaluated the expression of miRs in peripheral blood mononuclear cells (PBMC) of patients with and without MDD at baseline, and two and eight weeks after antidepressive treatments. The authors identified changes in several miRs (miR-107, miR-133a, miR-148a, miR-200c, miR-381, miR-425-3p, miR-494, miR-517b, miR-579, miR-589, miR-636, miR-652, miR-941, and miR-1243). Two of these miRs were overexpressed in MDD patients after an eight-week follow-up (miR-589 and miR-941). Furthermore, based on target profiling predicted for these miRs, a combination of four genes (*PPT1*, *TNF*, *IL1B* and *HIST1H1E*) showed potential as biomarkers that could have predictive value for treatment response [96].

Fan et al. [112] explored miRNAs expression in PBMC as specific blood-based biomarker for MDD patients, identifying 26 miRs with significant expression changes. After validating in a larger cohort, five miRs (miR-26b, miR-1972, miR-4485, miR-4498, and miR-4743) were found up-regulated, which would be controlling pathways associated with the nervous system and brain functions [112]. Wan et al. [113] examined the differential miRs expression profile in CSF and serum of MDD patients, finding three upregulated miRs (miR-221-3p, miR-34a-5p, let-7d-3p) and one repressed miR. These results were further validated in another 32 MDD patients. ROC analysis showed that the area under the curve (AUC) of let-7d-3p, miR-34a-5p, miR-221-3p and miR-451a was 0.94, 0.98, 0.97 and 0.94, with 90.48%, 95.24%, 90.48% and 90.48% specificity, and 93.75%, 96.88%, 90.63% and 84.85% sensitivity, respectively, suggesting that these miRs might serve as MDD biomarkers [113]. Most recently, Wang et al. [114] identified that miR-144-5p levels are associated with depressive symptoms, and miR-144-5p detection in plasma could be a potential biomarker for pathologic processes related to depression.

## 4. Epigenetics Modifications in MDD Therapy

In addition to being involved in depressive diseases physiopathology, epigenetic modifications could be implicated in the mechanism of action of antidepressants [115] (Table 4). Perisic et al. [116] reported that treatment with valproic acid may results in stronger global chromatin modifications, including histone H3/H4 hyperacetylation, 2MeH3K9 hypomethylation, and DNA demethylation [116]. In the same way, amitriptyline treatment can induce slight cytosine demethylation, paralleled by a reduction in DNA methyltransferase enzymatic activity, without affecting global histone acetylation status [116,117].

AD treatment can regulate epigenetic modifications affecting the expression of genes involved in MDD pathology. Electroconvulsive therapy (ECT) has been shown to be an effective and safe treatment for MDD patients [118]. However, the physiological mechanisms of ECT and its effects on brain structure are still unclear. In addition, some reports suggest the participation of histone modification in the mechanism of AD treatment. In this context, Tsankova et al. [119] found that histone modification controls the expression of *BDNF* after electroconvulsive stimulation in the brain hippocampus from rats (animal model equivalent of ECT), depending on treatment duration (30 min, two hours or 24 h), post-treatment time and gene promoter region. These results suggest that epigenetic modulation could be important for the action mechanism of ECT. Iga et al. [79] evaluated the expression of HDAC5 and cyclic AMP response element-binding protein 1 (CREB) in 20 MDD patients after eight-week paroxetine treatment. They reported higher HDAC5 and CREB post-treatment levels, and the correlation between levels of HDAC5 and CREB was positive [79]. Tsankova et al. [73] demonstrated that chronic social defeat stress induced lasting downregulation of Bdnf transcripts III and IV in mice hippocampus, and robustly increased repressive histone methylation at their corresponding promoters. Imipramine treatment in this model resulted in downregulation reversal and increased histone acetylation within these promoters. Hyperacetylation by chronic imipramine was associated with selective Hdac5 downregulation [73].

miRs have been also involved in the antidepressant mechanism of various drugs. At the moment, the first-choice therapy prescribed for patients suffering from MDD corresponds to a class of drugs known as Serotonin-selective reuptake inhibitors (SSRIs) [120]. SSRIs downregulate the serotonin transporter (SERT) and serotonin 1A (5-HT1A) autoreceptors on serotonergic neurons in the raphe nuclei. Nevertheless, to date, the stable modifications induced by chronic SSRI medication in serotonergic transmission is lacking a clear mechanism that can explain SERT and 5-HT1A repression [120]. Baudry et al. [121] demonstrated that SERT is a miR-16 target. When comparing miR-16 expression patterns, reports show higher levels of this miR in noradrenergic vs. serotonergic cells, where miR-16 repression in noradrenergic neurons can produce de novo SERT expression. Using a mice model, fluoxetine therapy has been shown to induce higher miR-16 levels within the serotonergic raphe nuclei, which is consequently followed by SERT repression [122,123]. Furthermore, raphe nuclei exposed to fluoxetine releases the neurotrophic factor S100b, which acts on noradrenergic cells of the locus coeruleus [121,123]. Based on these results, Baudry et al. [121] proposed that miR-16 contributes to the therapeutic action of SSRI antidepressants in monoaminergic neurons. In the same way, miR-1202 is a primate-specific miRNA associated with MDD pathophysiology and SSRIs responsiveness [124]. Issler et al. [125] identified a strong interaction between miR-135 and 5HT transporter and 5-HT1A receptor transcripts. Interestingly, miR-135a levels were up-regulated after AD treatment administration. Using genetically modified mice expressing higher or lower miR-135 levels, Issler and colleagues demonstrated major alterations in anxiety- and depression-like behaviors, 5HT levels, and behavioral response to AD treatment. Finally, miR-135a levels in blood and brain of depressed human patients were also evaluated, identifying significant lower expression levels. These results suggest both a potential role for miR-135 in the pathophysiology of MDD and its use as an endogenous antidepressant [125]. To date, few studies in human have been conducted to investigate the effect of AD treatment on epigenetic modifications. Some clinical trials have been performed (Table 4), but none has achieved significant results.

Independently to antidepressant treatment, it has shown that the use of epigenetic engineering can also be effective for treating patients with depression. Recently it has been shown that the use of zinc-finger proteins or transcription activator-like effectors, they can be used to control depression and addiction related behavior [126]. In the same way, Sun et al. demonstrate that the ATP-dependent chromatin remodeling can be a novel therapeutics targets in depressed patients to mediate depressive-like behavior [127]. These results represent a promising area of research in the treatment of patients with MDD.

## 5. Future Perspectives

The described studies indicate that psychiatric disorders, including MDD, are complex multifactorial diseases that include chronic alterations in the structure and function of neural circuits. Despite reports stating genetics plays an important role in the etiology of these diseases, there are discordances when analyzing the results of investigations on identical twins. These differences clarify the participation of additional factors. Environmental influences, like early life stress, play an important role in the development of psychiatric diseases to induce expression changes in important genes associated with MDD physiopathology. These changes would be mediated through epigenetic modifications, promoting or suppressing gene expression through three main mechanisms: DNA methylation, histone modification and miRs. Evidence supporting this hypothesis is large, and allows a better understanding of previously unknown physiopathological processes. In addition, the study of this new field suggests a possible link between the long-term effects of adverse life events and changes in gene expression associated with depression. Even though investigation of epigenetics in depression is still in development, numerous studies have hypothesized epigenetic modifications as potential biomarkers for depression diagnosis. It is expected that in coming years, epigenetic profiling will allow us to predict future susceptibility and/or MDD onset, improve diagnosis and to achieve a superior understanding on depression pathophysiology. Furthermore, epigenetics appears to be important for the mechanism of action of antidepressants. Future perspectives will aim to detect epigenetics modifications following AD therapy, which in turn will permit identifying new therapeutic targets based on epigenetics modifications for depressive diseases, ultimately helpful for monitoring treated patients.

## Figures and Tables

**Table 1:** DNA methylation studies in patients with depressive disorders.

Reference	Sample Characteristics	Study	Tissue	Diagnosis	Platform	Gene Associated	Potential Relevance of Gene in Depressive Disorders
Córdoba-Palomera et al. [49] 2015	17 MZ pairs Caucasian Spanish adult twins	Genome-wide DNA methylation	Peripheral blood	Anxious or depressive disorder	Illumina Infinium HumanMethylation450 Beadchip	*WDR26*	Prospective blood transcriptomic marker for depression [11,50].
						*CACNA1C*	Susceptibility factor for depressive psychopathology [11]. Methylation changes have been associated with risk factors for depressive disorders [51,52].
						*MAPK11*	Associated with depression phenotypes [53].
Sabunciyan et al. [54] 2012	39 individuals with MDD from Stanley Medical Research Institute	Genome-wide DNA methylation	Post-mortem frontal cortex	MDD	Comprehensive High-throughput Arrays for Relative Methylation (CHARM)	*PRIMA1*	Encodes a protein that functions to organize AChE into tetramers, and to anchor AChE to neural cell membranes [55,56].
Numata et al. [57] 2015	29 Medication-free patients with MDD	Genome-wide DNA methylation	Peripheral leukocytes	MDD	Infinium HumanMethylation450 BeadChips	*CAPRIN1*	Potential blood marker of major depressive disorder [58].
						*CITED2*	Differentially expressed in the mood disorder, associated with neurological or psychiatric diseases [40].
						*DGKH*	Risk gene for bipolar disorder [59,66].
Januar et al. [65] 2015	183 patients with MDD >65 years-old	High-throughput DNA methylation profiling	Buccal tissue	MDD	Sequenom MassARRAY	*BDNF*	Promotes the proliferation, differentiation and survival of neurons, crucial for neural plasticity and cognitive function [64]. Potential biomarker of depression [65].
Nieratschker et al. [38] 2014	8 mothers and their infants with prenatal stressed conditions. 9 pregnant rats with prenatal stressed conditions	Genome-wide association	Peripheral leukocytes and refrontal cortex of adult rats	MDD	Methylated DNA immunoprecipitation (MeDIP) and pyrosequencing	*MORC1*	Candidate gene for major depressive disorder related to early life stress in rodents, primates and humans [38]. Evokes a depression-like phenotype in mice [39].
Davies et al. [67] 2014	50 monozygotic twin pairs from the UK and Australia discordant for depression	Genome-wide DNA methylation	Whole blood and brain tissue samples	MDD	MeDIP-Sequencing	*ZBTB20*	Important for the hormonal hippocampal function, crucial for the regionalization and volume of archicortex, playing a role in depression [68,69].

MDD: Major depressive disorder; MZ: Monozygotic.

**Table 2:** Histone modification studies in patients with depressive disorders.

Reference	Sample Characteristics	Tissue	Diagnosis	Platform	Epigenetic Modification Evaluated	Gene and Histone Modification Associated	Main Findings
Cruceanu et al. [80] 2013	Individuals with bipolar disorder type I (*n* = 13) or MDD (*n* = 18) and controls (*n* = 14) with no psychiatric history	Post-mortem prefrontal cortex (PCF) from Broadman Area (BA) 10	BD or MDD	Chromatin immunoprecipitation (ChIP) and Quantitative real-time PCR	Histone modification	*SYN2* H3K4me3	H3K4me3 increase in MDD patients and correlated with gene expression of SYN2 [80].
Covington et al. [71] 2009	C57BL/6J male mice with chronic social defeat stress (*n* = 6) and control mice (*n* = 10). Patients depress postmortem (*n* = 8)	Brain tissue	Depression	Immunohistochemistry, Western blot and Illumina MouseWG-8 V2.0 array	Histone modification	H3K14ac	Transiently decreased and then stably increased of H3K14ac in the NAc of mice after chronic social defeat stress, correlated with a reduction in HDAC2 levels [71].
Hobara et al. [78] 2010	Mood disorder patients in a depressive and remissive state	Peripheral white cells	MDD and BD	Quantitative real-time PCR	Expression of HDACs	HDAC2 and HDAC5	Gene expression of HDAC2 and HDAC5 were significantly increased in MDD patients in depressive state compare to controls subjects, while during remissive state, HDACs expression was comparable to controls subjects, suggesting a state-dependent alteration [78].
Iga et al. [79] 2007	Patients diagnosed with MDD according to DSM-IV (*n* = 25) and controls (*n* = 25)	Peripheral leucocytes	MDD	Quantitative real-time PCR	Expression of HDACs	HDAC5	HDAC5 mRNA levels were significantly higher in drug-free depressive patients than controls [79].
Renthal et al. [77] 2007	Mice with chronic social defeat stress	Brain tissue	Depression	Immnunohistochemistry, ChIP and microarray	Histone modification and expression of HDACs	HDAC5	HDAC5 expression was decrease in a model with social defeat stress, imipramine treatment increased HDAC5 expression [77].

BD: Bipolar disorder; DSM-IV: Diagnostic and Statistical Manual of Mental Disorders IV.

**Table 3:** microRNAs (miRs) studies in patients with depressive disorders.

Reference	Sample Characteristics	Tissue	Diagnosis	Platform	miRNAs Associated	Main Findings
Uchida et al. [94] 2008	SH-SY5Y cells and Male rats Fisher 344 (F344) and Sprague-Dawley (SD) control with repeated restraint stress	Neuron cell lines Hypothalamic paraventricular nucleus	------	-----	miR-18a	Overexpressed in repeated restraint stress model. Its expression inhibits translation of the glucocorticoid receptor in neuron cell culture.
Vreugdenhil et al. [97,111] 2009	NS1 cells	Neuron cell lines	------	Luciferase reporter assay	miR-18a and miR-124a	miR-18a and miR-124a decrease protein expression of glucocorticoid receptor by luciferase reporter assay in NS1 cells.
Caputo et al. [101] 2011	HeLa cells	Cervix epithelial cell line	Schizophrenia	Luciferase reporter assay	miR-132 and miR-182	These miRNAs regulate the expression of BDNF by Allele-Specific Binding [101].
Smalheiser et al. [98] 2012	Antidepressant-free depressed suicide (*n* = 18) and well-matched non-psychiatric control subjects (*n* = 17)	Tissue, prefrontal cortex (Brodmann Area 9)	Depression	PCR miltiplex	miR-142-5p, miR-137, miR-489, miR-148b, miR-101, miR-324-5p, miR-301a, miR-146a, miR-335, miR-494, miR-20b, miR-376a*, miR-190, miR-155, miR-660, miR-130a, miR-27a, miR-497, miR-10a, miR-20a, miR-142-3p	miRs significantly downregulated in the prefrontal cortex of depressed patients compared with normal controls, many of them implicated in cellular growth and differentiation and some of them showed high synaptic enrichment [98,99].
Belzeaux et al. [96] 2012	16 severe MDE patients and 13 matched controls	Peripheral blood mononuclear cells	Major depressive episode	Microarray SurePrint G3 Human GE 8 x 60 K	miR-107, miR-133a, miR-148a, miR-200c, miR-381, miR-425-3p, miR-494, miR-517b, miR-579, miR-589, miR-636, miR-652, miR-941, miR-1243	miRs significantly deregulated between MDE patients and controls. These miRs help finding a gene combination useful to predict treatment response [96].
Bocchio-Chiavetto et al. [100] 2013	10 patients with MD, the sample was extracted before and after treatment	Blood	MDD	TaqMan Array Human MicroRNA A + B Cards Set v3.0	**UP**: miR-130b*, miR-505*, miR-29b-2*, miR-26b, miR-22*, miR-26a, miR-664, miR-494, let-7d, let-7g, let-7e, let-7f, miR-629, miR-106b*, miR-103, miR-191, miR-128, miR-502-3p, miR-374b, miR-132, miR-30d, miR-500, miR-589, miR-183, miR-574-3p, miR-140-3p, miR-335, miR-361-5p. **DOWN**: miR-34c-5p and miR-770-5p	Associated with neuronal brain function, such as neuroactive ligand–receptor interaction, axon guidance, long-term potentiation and depression [100].
Li et al. [33] 2013	40 patients and 40 healthy controls	Serum	MDD	Real time PCR	miR-132 and miR-182	The expression of these miRs was negatively correlated with BDNF expression [33].
Fan et al. [112] 2014	81 MDD patients and 46 healthy controls	Peripheral blood mononuclear cells	MDD	Affymetrix miRNA 3.0 array	miRNA-26b, miRNA-1972, miRNA-4485, miRNA-4498, and miRNA-4743	Overexpressed in MDD patients, and would regulate pathways associated with nervous system and brain functions [112].
Wan et al. [113] 2015	1° cohort: 6 depressed and 6 control patients. 2° cohort: 32 MDD patients and 21 healthy individuals	Peripheral blood mononuclear cells	MDD	microRNA PCR Panel (V3.M)	let-7d-3p, miR-34a-5p, miR-221-3p, miR-451a	Potential MDD biomarkers [113].
Wang et al. [114] 2015	169 patients and 52 controls	Plasma	Depression	Serum/Plasma Focus microRNA PCR Panel	miR-144-5p	miR-144-5p levels are associated with depressive symptoms, and the detection of this miR in plasma could be a potential peripheral biomarker for pathologic processes related to depression [114].

MDE: Major depressive episode.

**Table 4:** Clinical trials of antidepressant treatment associated epigenetic modifications.

Study	ClinicalTrials.gov Identifier	Status	Phase	Aims	Intervention	Condition	Publications
Paliperidone and lithium in the treatment of suicidality—treatment indication and epigenetic regulation (AFSP)	NCT01134731	Completed	Phase 4	To identify an efficient pharmacotherapy for the acute management of suicidality and the epigenetic regulation associated with the treatment.	Paliperidone and lithium	MDD Suicidality	Not provided
Epigenetic regulation of brain-derived neurotrophic factor (BDNF) in major depression	NCT01182103	Completed	-----	To detect the associations between BDNF, DNA methylation, histone modification, depressive symptoms, suicidal behavior and antidepressant responses in MDD patients, check the correlation between blood BDNF protein and RNA and BDNF rs6265 gene, and discuss the possible mechanisms of epigenetic regulation of BDNF in Taiwanese MDD patients.	-----	MDD	Not provided
A neuroimaging and epigenetic investigation of antidepressants in depression	NCT00703742	Completed	-----	To find out the structural or functional effects of SSRI in MDD; to explore the DNA methylation status in depression; to find special abnormalities in depression secondary to other disease (autoimmune disease like systemic lupus erythematosus).	Escitalopram	Depression secondary to other disease	[128,129]
miRNAs, suicide, and ketamine—plasma exosomal microRNAs as novel biomarkers for suicidality and treatment outcome	NCT02418195	Recruiting participants	Phase 2	To examine whether neural-derived exosomal miRNAs are differentially expressed that are specific to suicidal ideation or behavior, and which by affecting specific miRNA targets and pathways, are associated with suicidal behavior and response to ketamine.	ketamine	MDD	Not provided
Add-On Study of MSI-195 (*S*-adenosyl-l-methionine, SAMe) for patients with major depressive disorder (MDD)	NCT01912196	Ongoing	Phase 2	To determine the efficacy and safety of 800 mg MSI-195 in reducing symptoms of depression in Major Depressive Disorder (MDD) patients with inadequate response to current antidepressant therapy.	MSI-195 and Placebo	MDD	Not provided
